# Comparing residential contamination in a Houston environmental justice neighborhood before and after Hurricane Harvey

**DOI:** 10.1371/journal.pone.0192660

**Published:** 2018-02-08

**Authors:** Jennifer A. Horney, Gaston A. Casillas, Erin Baker, Kahler W. Stone, Katie R. Kirsch, Krisa Camargo, Terry L. Wade, Thomas J. McDonald

**Affiliations:** 1 Department of Epidemiology and Biostatistics, Texas A&M School of Public Health, College Station, Texas, United States of America; 2 Interdisciplinary Program in Toxicology, Texas A&M University, College Station, Texas, United States of America; 3 Pacific Northwest National Laboratory, Richland, Washington, United States of America; 4 Geochemical and Environmental Research Group, Texas A&M University, College Station, Texas, United States of America; 5 Department of Environmental and Occupational Health, Texas A&M School of Public Health, College Station, Texas, United States of America; Shantou University Medical College, CHINA

## Abstract

**Introduction:**

Polycyclic aromatic hydrocarbons (PAHs) are complex environmental toxicants. Exposure to them has been linked to adverse health outcomes including cancer, as well as diseases of the skin, liver, and immune system. Based on an ongoing community engagement partnership with stakeholder groups and residents, we conducted a small longitudinal study to assess domestic exposure to PAHs among residents of Manchester, an environmental justice neighborhood located in the East End of Houston, TX.

**Methods:**

In December, 2016, we used fiber wipes to collect samples of household dust from 25 homes in Manchester. Following Hurricane Harvey, in September 2017, we revisited 24 of the 25 homes to collect soil samples from the front yards of the same homes. Wipes and soil were analyzed for the presence of PAHs using gas chromatography–mass spectrometry (GC-MS) methods. Principal component analysis plots, heatmaps, and PAH ratios were used to compare pre- and post-Hurricane Harvey samples.

**Results:**

While direct comparison is not possible, we present three methods for comparing PAHs found in pre-hurricane fiber wipes and post-hurricane soil samples. The methods demonstrate that the PAHs found before and after Hurricane Harvey are likely from similar sources and that those sources are most likely to be associated with combustion. We also found evidence of redistribution of PAHs due to extreme flooding associated with Hurricane Harvey.

**Discussion:**

Residents of the Manchester neighborhood of Houston, TX, are exposed to a range of PAHs in household dust and outdoor soil. While it was not possible to compare directly, we were able to use several methods to assess detected concentrations, changes in site-specific PAH allocations, and PAH origination. Additional research is needed to identify specific sources of domestic PAH exposure in these communities and continued work involving community members and policy makers should aim to develop interventions to reduce domestic exposure to and prevent negative health outcomes from PAHs.

## Introduction

Over the last decade, calls to increase the quantity and improve the quality of post-disaster research have come from both the academic and applied public health communities [1–4]. Rapid and credible post-disaster research is now seen as essential for protecting the health of the public and responders, and necessary for increasing trust of governmental response agencies among affected populations [5]. Conducting meaningful research in the context of a public health emergency requires addressing several potentially challenging gaps including: knowledge gaps presented by public health emergencies that require interdisciplinary and academic-practice collaborations to address; difficulties in planning for, and rapidly executing, scientific research in the context of disaster response; and a general lack of available baseline data to compare with post-disaster findings to determine changes that may be attributable to the disaster [3,5]. This paper offers one approach to addressing these three challenges by presenting results from a small study of pre- and post-Hurricane Harvey exposure to polycyclic aromatic hydrocarbons (PAHs) among residents of a Houston environmental justice neighborhood.

Due to the variety and complexity of both direct and indirect adverse impacts of disasters, the assessment of the public health impacts of disasters and emergencies requires the expertise of a wide range of field- and laboratory-based scientists [6]. While early disaster research was predominantly applied, descriptive, and based on self-reported data, more recently disaster research has provided the setting for theoretical and methodological innovations [7,8] as well as hypothesis-oriented research designs supported with biologic and environmental sampling [9,10]. Partnerships with emergency management, community stakeholders, and decision makers are also necessary to ensure that research is responsive to community concerns and can be used to guide relief activities and policy making [11].

To successfully conduct research in the aftermath of a disaster, protocols and other documents must be in place before the disaster. Disaster research, although it must be initiated quickly, must not compromise or interfere with response and recovery efforts, cause additional physical or mental stress to those who have been impacted, or disregard the balance of burdens and benefits required during the review of research ethics [12,13]. Following the 2010 Deep Water Horizon oil spill, a national disaster research response program was established by the National Institutes of Environmental Health Sciences to create tools, protocols, and research networks that could be activated quickly to assess the environmental health impacts of a disaster [5]. One outcome from this effort was the establishment of a central Institutional Review Board (IRB) at the National Institutes of Health to provide timely reviews of multiagency studies involving human participants [2]. More work is needed to ensure university [14,15] and health department [16] IRBs understand and are able to act on protocols for disaster research quickly enough to get researchers to the field. Obtaining funding to support post-disaster research can also be challenging, with few response grants being truly “rapid” in the sense of supporting immediate deployment of researchers to the field [17,18]. Study designs and statistical methods may also need to be adapted from those commonly used in more traditional epidemiologic studies [19]. For example, difficulties in characterizing populations at risk may lead to exposure misclassification, since exposure data is frequently not available at smaller spatial scales. Disaster research frequently uses disaster declarations issued by the Federal Emergency Management Agency (FEMA) at the county level, although neither impacts nor susceptibility are likely distributed equally at this level [20].

Another principal limitation of disaster research is the availability of sound baseline data, which is often lacking. Pre-disaster baseline data are rarely available for populations or environments impacted by disasters. Studies of the mental health impacts of the World Trade Center disaster used baseline assessments conducted one year after the attack, with follow-up assessment conducted two years after the attack [21]. In a review of 225 disaster mental health studies conducted by Norris (2006), the majority were cross-sectional, after-only study designs [22]. While studies that included both pre- and post-disaster data reported less severe outcomes than those with post-disaster data alone, the populations that were evaluated in these studies were selected due to the availability of pre-disaster baseline data, rather than the severity of disaster impact [22].

The tandem growth of disaster frequencies and populations living near industrialized areas has brought greater attention to the potential health effects of environmental contamination associated with joint natural and technological (na-tech) disasters [23,24,25,26]. Na-tech events occur as a result of disaster-associated technological malfunction or failure, leading to the unintentional release of hazardous materials [25]. After Hurricane Floyd, more than 50 large-scale hog waste lagoons in North Carolina were inundated or breached, contaminating nearby waterways with animal waste [27]. Following the Indian Ocean Tsunami of 2004, sewage overflow, chemical releases from farms and factories, and hazardous medical and household chemical debris threatened the quality of ground and surface water [28]. In the aftermath of Hurricane Harvey, potential environmental hazards resulting from chemical plant explosions [29], flooding of a designated Superfund site [30], and toxic emissions from a refinery located in the Manchester neighborhood [31] were documented by the media.

PAHs are primarily formed from the incomplete combustion of organic materials, sources of which include biofuel and petroleum consumption, wildfires, and consumer products [32,33,34]. In 1976, the U.S. Environmental Protection Administration (EPA) classified 16 PAHs as priority pollutants [32] due to their known toxicity to humans, occurrence in the environment, and the analytical capabilities that were available at the time [35]. Seven of these priority pollutants–designated by the EPA as Group B2 –are classified as reasonably anticipated to be human carcinogens, including (benzo(a)anthracene, 5-methylchrysene, benzo(a)pyrene, benzo(b)fluoranthene, benzo(k)fluoranthene, dibenz(a,h)anthracene, and indeno(1,2,3-cd)pyrene) [36]. Although the PAHs identified as being priority pollutants have been used to develop regulatory standards, advances in research and analytical capabilities have identified a need to investigate the potential human health and environmental impacts of additional PAH compounds [37].

The human health impacts of PAHs are dependent upon the toxicity and concentration of the compounds to which an individual is exposed, as well as the period of exposure (chronic or acute), route of exposure (inhalation, dermal contact, or ingestion), and individual immunological capacity [32]. While the literature describing adverse health outcomes associated with PAHs is largely focused on occupational exposures [38,39], a growing body of evidence suggests that residential settings may also serve as important environmental reservoirs of these compounds [40,41,42]. Although the health impacts of short-term PAH exposures are not well understood [43], exposure to background PAH concentrations has been associated with elevated serum markers of inflammation [44]. Long-term occupational exposures has been shown to increase the risk of developing certain cancers, including cancers of the breast, lung, gastrointestinal, and bladder cancers [38,39,45,46]. According to the estimates derived by Zhang et al. (2016), in 2011, there were 5,704 excess lifetime cases of cancer in the U.S. as a result of ambient PAH exposures [47]. Children are inherently more sensitive than adults to PAH exposures because they are likely to ingest greater amounts, measured in milligrams by body weight, and their organ and immune systems are not fully developed [48]. Hand-to-mouth activity during play on household floors can expose toddlers to 2.5 times greater PAH levels relative to adults [49]. Prenatal exposure to excess levels of airborne PAHs has been linked to physical impairments such as impaired fetal growth [50,51], congenital malformations [52,53,54,55] and deficits in developmental milestones [56,57].

## Materials and methods

### Ethics statement

The materials associated with this project were reviewed and approved by the Texas A&M University Institutional Review Board (IRB 2016-0698D). All participants provided informed consent by signing a copy of a consent form approved by the IRB after receiving information about participation.

### Study location

**Fig 1:**
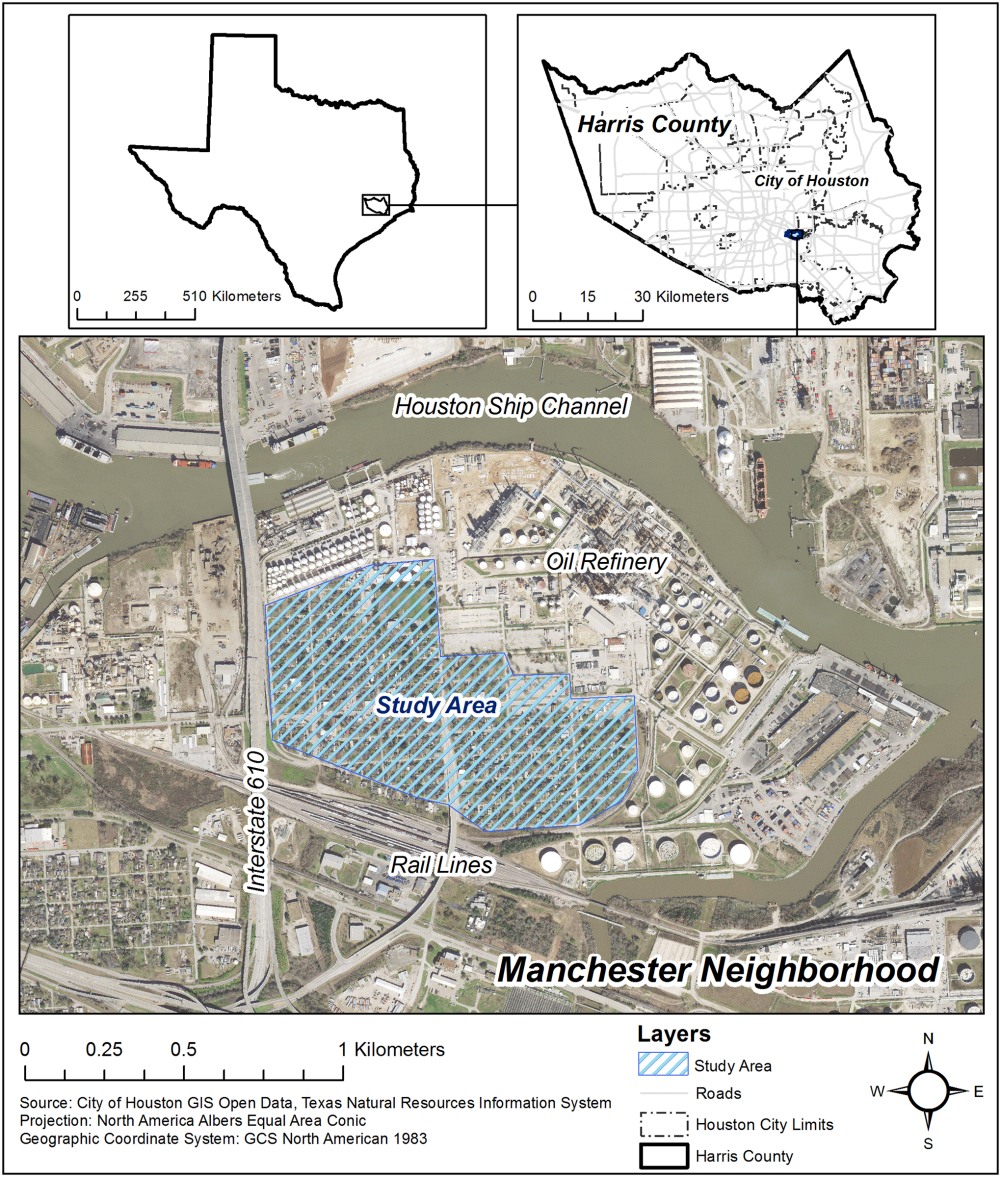
Manchester neighborhood, Houston, Texas.

Environmental justice communities are made up of low income, minority individuals and groups who are at disproportionate risk from the health and other impacts of exposure to pollution and environmental stressors in their homes, neighborhoods, and workplaces [58]. Numerous studies have illustrated the health inequities associated with the siting of environmentally hazardous industries, including a quarter of the refining capacity of the U.S. and more than 200 petro-chemical facilities, along Houston’s Ship Channel [59,60,61,62]. Communities located along the Houston Ship Channel and Sims Bayou, including the neighborhood of Manchester, are at particularly high risk for the health impacts from the nexus of exposure to hazardous substances and natural disasters during na-tech events (Fig 1). Within 1 mile of the Manchester neighborhood, there are 21 facilities that report to the EPA’s Toxic Release Inventory: 11 large-quantity generators of hazardous waste; 4 facilities that treat, store, or dispose of hazardous wastes; 9 major dischargers of air pollution; and 8 major storm water discharging facilities [63]. The area is also highly vulnerable to the impacts of natural disasters, both socially and physically. The population of the Harrisburg/Manchester Super Neighborhood is 90% Hispanic and 6% African-American, with a median income that is one-third less that the City of Houston overall [64]. Floodplains along Sims Bayou have increased by 15% since 1980, due to increases in development and impervious cover like concrete and asphalt, while sea-level rise could expose another 35,000 residents in Houston Ship Channel and Sims Bayou neighborhoods to regular flooding by 2050 [65].

As part of a pilot project funded by the Texas OneGulf Center of Excellence, a team of researchers at the Texas A&M Institute for Sustainable Communities have been working with Texas Environment Justice Advocacy Services (t.e.j.a.s.) and residents of the Manchester neighborhood since December, 2016, to collect survey data and environmental samples to characterize residents’ exposure to heavy metals, volatile organic compounds, and PAHs in indoor dust and outdoor surface soil. This ongoing study meant that the study team had an established partnership with residents of Manchester and t.e.j.a.s., an advocacy group that represents their concerns about environmental contamination, as well as baseline data on hand that could be compared to samples collected at the request of t.e.j.a.s. and local residents after Hurricane Harvey made landfall along the Texas Coast as a Category 4 storm on August 25, 2017 [66]. The wettest tropical cyclone on record, Hurricane Harvey dumped up to 64.5 inches of rain in Houston–a metropolitan area larger than New Jersey with 6 million residents and >20 Superfund sites–potentially exposing residents to a variety of chemicals and toxins [67,68,69]. On September 1, 2017, the research team, accompanied by community partners, revisited homes in Manchester to collect soil samples for analysis to assess potential environmental health effects of Hurricane Harvey.

### Sample collection

Using Geographic Information Systems ArcMap (ESRI, Redlands, CA, USA), 25 homes in the geographically compact Manchester neighborhood of Houston, Texas, were selected and settled household dust samples were collected in November, 2016 (West: -95.265927; East: -95.252709; North: 29.722526; South: 29.714624) (Fig 1). A team comprised of a Texas A&M researcher and staff at t.e.j.a.s. approached each selected home and used a particulate wipe to collect settled dust from non-carpeted flooring (tile, wood, or linoleum) located in the entryway of each home. If the home was completely fenced off or abandoned, or if the resident refused to participated, the next nearest home was approached. The cooperation rate (number of completed samples divided by housing units contacted) was 61% (25/41). Each team measured and marked a 1 m^2^ area within each home and took one sample. After aseptically donning powder-free vinyl gloves, each team removed a prepared glass fiber filter cloth from an aluminum foil protective wrap. Approximately 5 mL of 100% isopropyl alcohol was applied to the cloth and the floor was cleaned starting at the top of the marked area and moving across and down in one continuous motion. The fiber cloth was then returned to the foil container, placed into a sterile and labeled polypropylene plastic resin bag, and placed into a cooler for transport to a laboratory at the Texas A&M School of Public Health. Upon arrival at the laboratory, samples were stored at -4°C prior to extraction and analysis. These methods have been utilized and well described previously [70,71].

In September of 2017, soil samples were collected from the front yards of the same homes in Manchester. The latitude and longitude of each sampling site was recorded. After aseptically donning powder-free vinyl gloves, each team used a small, metal hand trowel to collect the superficial layer of soil (0–3 cm). Each sample was deposited into a sterile, labeled 16 oz. prepared glass jar. Samples were transported to the Geological and Environmental Research Group (GERG) laboratory at Texas A&M University and stored at -20°C.

### PAH extraction

To recover the organic material from the dust samples, each filter cloth was processed by pressurized fluid extraction in a Dionex ASE 200 Accelerated Solvent Extractor using a modified U.S. EPA Method 3545A [72]. Soil samples were removed from storage and held at room temperature until thawed. A 20 g subsample was aseptically transferred into a sterile glass jar and then treated with 15–20 g of anhydrous sodium sulfate. Extraction cells were prepared by inserting combusted filters, followed by granular copper activated by combining 38% hydrochloric acid. Next, the prepared sample was transferred into the cell, with blank cells prepared first. Finally, 100 ml of surrogate was transferred into each cell with a micropipette, which was rinsed in methylene chloride at least five times between each sample. Cell caps were rinsed with methanol/methylene chloride and then screwed onto cells.

The organic compounds present in the soil samples were extracted using an accelerated solvent extractor (ASE 200, Dionex) with dichloromethane. The soil were extracted under high-temperature (100°C) and high pressure (1500 psi). The extract of each sample was filtered through a sodium sulfate filtration funnel and transferred into a 250 mL volumetric flask. Next, samples activated, granular copper (20–30 mesh) and boiling chips were transferred to the flask. The sample extracts were concentrated by evaporation; prepared flasks containing the samples were transferred into a water bath previously heated to 60°C. The samples were concentrated to a final volume of 1–2 ml. Finally, the solvent was exchanged with hexane by slowly adding small amounts of hexane to the extract while the sample was being concentrated and then the sample was purified using column chromatography.

### GC-MS analysis

Fiber cloth and soil extracts were analyzed for PAH hydrocarbon concentrations using a HP5890 gas chromatography system (HP5890, Hewlett Packard Company, Wilmington, DE) coupled with an Agilent 5972 mass spectrometer (Agilent 5972, Agilent Technologies, Santa Clara, CA) for GC-MS measurements [73]. A HP-5MS capillary column (Agilent HP-5MS 60 m long with an interior diameter of 0.25 mm and 0.25 μm film thickness, Agilent Technologies, Santa Clara, CA) was used to chromatographically separate the PAH analytes. The initial temperature of the injection port was held at 285°C, vaporizing the injected extract prior to entering the capillary column. The oven was initially set to 60°C. After injection, the oven was programmed to increase in temperature at a rate of 7°C/min until it reached the final holding temperature of 310°C with a final holding time of 22 minutes. Selected ion monitoring (SIM) mode was used to identify and quantify PAH components. The use of SIM enables the determination of analytes of interest and improves the ability to measure highly specific compounds that occur at lower concentrations within the extract (McDonald et al. 2000). Data generated with the GC-MS platform were quantified using the ChemStation program (ChemStation software, Agilent Technologies, Santa Clara, CA).

To establish retention times, organics in marine sediment standard reference material (SRM-1941b), were used. The GC-MS measurements included: a system tune, six-level initial calibration (ICal), independent calibration verification solution (ICV), continuing calibration checks (CCC), reference oil (SRM 1582), method blank, blank spike (BS), and blank spike duplicate (BSD). PAH analytes were quantified, including the EPA’s 16 priority PAHs (e.g., benzo(a)anthracene, benzo(a)pyrene, benzo(b)fluoranthene, benzo(k)fluoranthene, chrysene, dibenzo(a)anthracene, and indeno(1,2,3-c,d)pyrene, acenaphthene, acenaphthylene, anthracene, benzo(g,h,i)perylene, fluoranthene, fluorene, naphthalene, phenanthrene, and pyrene [72]. The practical quantitative limit for each analyte was 10 ng/mg extract.

### Data analysis

The amount of PAHs in the wipes and soils was assessed by evaluating the total mass observed versus the sampling area (μg/m^2^ or μg/kg). All concentrations were log transformed and analyzed with a 3-dimensional principal component analysis (PCA) plot using MetaboAnalyst 3.0 (Montreal, Canada) to understand sample similarities. A PCA diagnostic isomer ratio analysis of anthracene to anthracene plus phenanthrene (A/A+P) and fluoranthene to fluoranthene plus pyrene (F/F+Py) were used to determine the potential origin of the PAHs. As shown by Yunker et al. (2002), the diagnostic ratios can be used to indicate either combustion or other origins for the PAHs [74].

## Results

**Fig 2:**
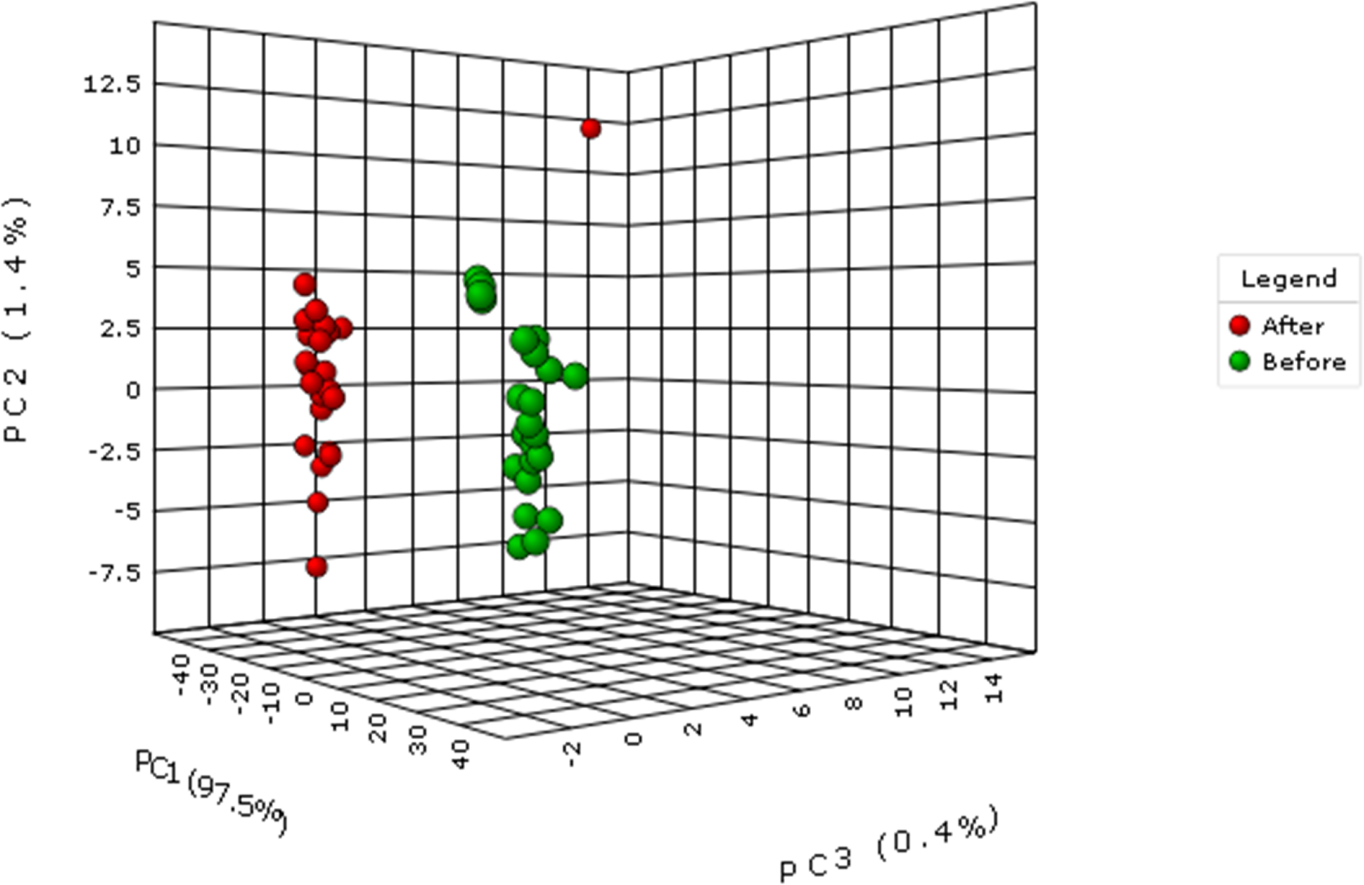
Three-dimensional principal component analysis (PCA) plot illustrating how the data from each site and collection type clustered. Soil sites are shown in red; wipes are shown in green. Before Hurricane Harvey: Green; After Hurricane Harvey: Red.

The concentration of total PAHs in each wipe sample was widely variable, ranging from 0.29 to 3.95 μg/m^2^. The concentration of total PAHs in each soil sample ranged from 54.65 to 4,378.25 μg/kg. From these ranges, it was observed that the PAH concentrations were very different in the wipes and soils. To further evaluate the sample types, the concentrations were log transformed then analyzed by PCA. In the PCA, the wipes grouped together and the soils were also clustered, except for one site (Fig 2).

Analysis of the raw data from the soil that did not cluster showed that it was the only site where dibenzo(a,h)anthracene was not detected following the hurricane. However, in the wipe sample collected from the same site prior to the hurricane, dibenzo(a,h)anthracene was observed, illustrating that Hurricane Harvey redistributed this PAH from its location. Average concentration values for PAHs detected in the wipe and soil samples are shown in Table 1. A supporting table shows all 16 priority PAH values for all sampling locations for both wipes and soil (S1 Table).

**Table 1:** PAH concentrations in wipes and soil, Manchester, Houston, TX.

	Pre-Hurricane Harvey Wipes (μg/m^2^)	Post-Hurricane Harvey Soil (μg/kg)
Acenaphthene	0.035	548.000
Acenaphthylene	0.016	90.388
Anthracene	0.033	152.013
Benz(a)anthracene	0.024	578.092
Benzo(a)pyrene	0.022	616.471
Benzo(b)fluoranthene	0.052	1117.896
Benzo(g,h,i)perylene	0.016	0.090
Benzo(k,j)fluoranthene	0.028	256.950
Chrysene/Triphenylene	0.071	827.763
Dibenzo(a,h)anthracene	0.005	108.713
Fluoranthene	0.095	1020.925
Fluorene	0.031	230.463
Indeno(1,2,3-c,d)pyrene	0.013	512.554
Naphthalene	0.083	868.946
Phenanthrene	0.123	453.238
Pyrene	0.075	853.088

**Fig 3:**
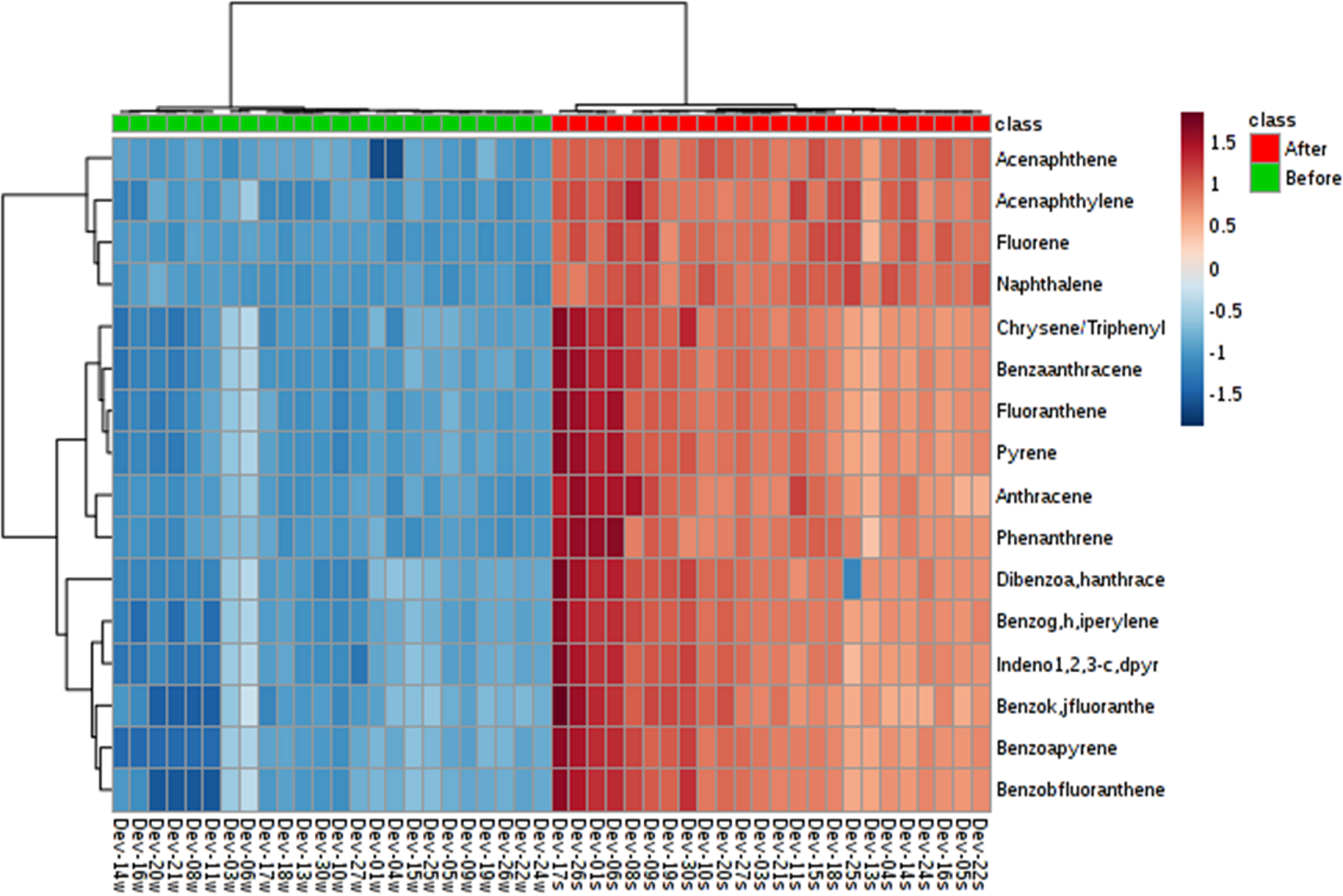
A heatmap of PAH concentrations for all wipe and soil locations. Soil sites are shown in red for their class; wipes are shown in green. The scale from 1.5 to -1.5 illustrates whether a certain site has a higher or lower PAH concentration in comparison to all sites.

To further study this redistribution, we also prepared a heatmap of all values (Fig 3). The heatmap showed that after Hurricane Harvey, the relative order of PAH concentrations across sampling locations was different. The sites with higher levels of pre-hurricane PAHs, relative to other locations, had lower levels after the hurricane and vice-versa. Specifically, benzo(b)fluoranthene was undetected in the wipes from Sample 8, while it had one of the higher levels in the soil samples. Sample 3 also had one of the highest levels for many of the PAHs prior to the hurricane, when compared to the other sites, but following the hurricane its levels were more on the average range, showing dilution of the PAHs. Many other examples of this can be observed, again indicating that PAHs were redistributed following the huge amount of water resulting from precipitation due to Hurricane Harvey.

Finally, to assess the potential sources of the PAHs, we calculated the ratios of A/A+P and F/F+Py for both pre-Hurricane Harvey wipes (Table 2) and post-Hurricane Harvey soil samples (Table 3). Due to the location of the sites in this study (e.g., Manchester is bordered by a major interstate highway, a 24 line railyard, and a large refinery complex), we expected the main source of PAHs to be combustion. One way to evaluate this was developed by Yunker et al. (2002), who demonstrated A/A+P ratio values <0.10 are indicative of a petroleum source, while values >0.10 indicate combustion as the dominant source [74]. Likewise, F/F+Py ratios <0.40 indicate a petroleum source, while values >0.40 indicate combustion as the dominant source. These data indicate that the dominant sources of PAHs present in both pre- and post-hurricane samples are from combustion.

**Table 2:** Pre-Hurricane Harvey PAH ratios, anthracene/anthracene plus phenanthrene (A/A+P) and fluoranthene/fluoranthene plus pyrene (F/F+P) (μg/m^2^).

Sample	Anthracene	Phenanthrene	A/A+P	Fluoranthene	Pyrene	F/F+P
1	0.037	0.235	0.136	0.091	0.063	0.589
2	0.086	0.288	0.229	0.276	0.231	0.544
3	0.017	0.086	0.165	0.039	0.044	0.473
4	0.039	0.128	0.235	0.139	0.103	0.574
5	0.140	0.325	0.301	0.750	0.541	0.581
6	0.029	0.130	0.181	0.041	0.0358	0.535
7	0.038	0.112	0.252	0.064	0.051	0.554
8	0.023	0.087	0.211	0.023	0.020	0.533
9	0.028	0.144	0.163	0.093	0.074	0.558
10	0.020	0.098	0.170	0.038	0.034	0.527
11	0.019	0.081	0.192	0.017	0.017	0.494
12	0.044	0.070	0.386	0.066	0.055	0.547
13	0.023	0.087	0.211	0.026	0.024	0.523
14	0.029	0.166	0.150	0.103	0.063	0.620
15	0.020	0.075	0.213	0.043	0.037	0.539
16	0.024	0.094	0.201	0.070	0.073	0.489
17	0.020	0.068	0.224	0.017	0.016	0.510
18	0.018	0.065	0.212	0.022	0.013	0.622
19	0.018	0.092	0.162	0.071	0.054	0.566
20	0.0228	0.102	0.179	0.064	0.057	0.531
21	0.024	0.104	0.187	0.070	0.067	0.514
22	0.021	0.073	0.220	0.053	0.045	0.540
23	0.037	0.134	0.216	0.043	0.037	0.540
24	0.025	0.111	0.184	0.053	0.038	0.581

**Table 3:** Post-Hurricane Harvey PAH ratios, anthracene/anthracene plus phenanthrene (A/A+P) and fluoranthene/fluoranthene plus pyrene (F/F+P) (μg/kg).

Sample	Anthracene	Phenanthrene	A/A+P	Fluoranthene	Pyrene	F/F+P
1	360.7	1892.3	0.160	2442.4	1750.5	0.583
2	30.1	117.8	0.204	239.3	195.1	0.551
3	29.4	78.6	0.272	145.1	131.3	0.525
4	14.0	74.0	0.159	116.0	95.4	0.549
5	407.3	2674.8	0.132	4290.6	2984.3	0.590
6	411.8	112.8	0.785	390.0	468.9	0.454
7	117.4	264.0	0.308	485.4	392.1	0.553
8	30.0	95.1	0.240	219.5	185.5	0.542
9	140.2	195.1	0.418	312.0	321.6	0.492
10	8.9	22.0	0.288	43.8	37.0	0.542
11	41.6	105.3	0.283	104.8	90.4	0.537
12	64.6	234.5	0.216	231.1	188.1	0.551
13	18.0	75.3	0.193	89.4	79.6	0.529
14	315.8	1641.2	0.161	8145.1	6909.2	0.541
15	37.4	203.8	0.155	140.2	113.4	0.553
16	58.6	197.7	0.229	455.3	373.8	0.549
17	32.1	113.5	0.220	282.8	238.3	0.543
18	33.2	139.2	0.193	244.4	193.8	0.558
19	14.5	69.1	0.173	139.9	127.3	0.524
20	23.7	79.8	0.229	173.0	143.0	0.547
21	20.1	123.3	0.140	71.4	59.5	0.545
22	701.5	2091.3	0.251	5054.1	4600.6	0.523
23	54.9	184.0	0.230	379.3	313.4	0.548
24	682.5	93.2	0.880	307.3	482.0	0.389

## Discussion

The concentration of PAHs in urban soils located in close proximity to residential structures may increase the risk of inhalation, ingestion, or dermal contact exposures to these pollutants [75]. Therefore, information describing the concentrations and sources of PAHs in domestic dust and soils is important as part of the development of interventions to reduce potential adverse health outcomes among exposed populations. Since the health impacts of exposure to PAHs are potentially serious and little is known about the health impacts of domestic exposures, a study intended to characterize domestic exposure to PAHs and other contaminants among residents of an environmental justice community located in Houston, TX, was initiated by Texas A&M University and community partners t.e.j.a.s. in December 2016, which provided baseline data for the current study. After the collection of this data, this area was impacted by Hurricane Harvey, providing an opportunity to assess the potential for PAHs to be redistributed after a major flooding event.

In general, one major limitation of the post-disaster assessment of environmental exposures is the lack of baseline exposure data. In this small pilot study, we were able to compare pre-Hurricane Harvey PAHs measured in filter wipes and post-Hurricane Harvey PAHs measured in soil samples. While direct comparison is not possible, we were able to use several methods to assess detected concentrations, changes in site-specific PAH allocations, and PAH origination. For example, GC-MS allowed for the evaluation of PAH concentrations at each sampling site. PCA demonstrated similarities between collection types, while heatmaps illustrated the redistribution of PAHs across sampling locations. Diagnostic ratios consistently demonstrated combustion sources. These findings are an important first step towards demonstrating the potential for acute post-disaster environmental exposures to be additive to ongoing chronic environmental exposures.

This study has several important limitations. Pre-hurricane samples were collected as part of a small pilot cohort study that included the collection of environmental samples using filter wipes from inside homes located in the Manchester neighborhood. This study also included a cross-sectional assessment of potential PAH sources (e.g., indoor smoking), as well as the collection of indoor air (N = 11; preliminary results showed multiple volatile organic compounds present in the samples but not in the standard) and tap water (N = 25; no contaminants detected). While this paper does not report results from adjusted models to control for factors such as smoking, no residents reported smoking in their homes at the time of the pre-Hurricane data collection. Due to time constraints and access concerns (e.g., many roads remained flooded until the day before we collected samples) and to minimize the burden to participants due to the catastrophic impacts of Hurricane Harvey on Houston residents, soil samples collected after Hurricane Harvey were collected in the yard of the same homes to eliminate the need for residents to be at home to provide access for indoor sampling. In addition, the soil samples allowed us to assess the effects of flood water, which was generally present only in yards and not inside homes in this particular area of Houston [76]. However, these small, older homes (average square feet = 1,000; most built between 1930 and 1949) do not have garages [77]), so it is typical for residents to walk through their yards to enter their homes.

## Conclusion

Our small pre-post Hurricane Harvey study demonstrated redistribution of PAHs in an environmental justice neighborhood located in Houston, TX. While this study is small, and direct comparison is not possible due to the different collection methods, the unique ability to compare pre- and post-Hurricane sampling locations enabled an understanding of molecular changes following disaster conditions. Since pre-disaster samples are rarely available for comparison, this study advances our understanding of the potential environmental health impacts of disasters.

## Supporting information

S1 Table Total PAHs present in fiber wipes and soil samples at each sampling location. (XLSX)
